# The intracellular angiotensin system buffers deleterious effects of the extracellular paracrine system

**DOI:** 10.1038/cddis.2017.439

**Published:** 2017-09-07

**Authors:** Begoña Villar-Cheda, Maria A Costa-Besada, Rita Valenzuela, Emma Perez-Costas, Miguel Melendez-Ferro, Jose L Labandeira-Garcia

**Affiliations:** 1 Laboratory of Neuroanatomy and Experimental Neurology, Department of Morphological Sciences, Faculty of Medicine, CIMUS, University of Santiago de Compostela, Santiago de Compostela, Spain; 2 Networking Research Center on Neurodegenerative Diseases (CIBERNED), Spain; 3 Division of Pediatric Nephrology, Department of Pediatrics, University of Alabama at Birmingham, Birmingham, AL, USA; 4 Division of Pediatric Surgery, Department of Surgery, University of Alabama at Birmingham, Birmingham, AL, USA

## Abstract

The ‘classical’ renin–angiotensin system (RAS) is a circulating system that controls blood pressure. Local/paracrine RAS, identified in a variety of tissues, including the brain, is involved in different functions and diseases, and RAS blockers are commonly used in clinical practice. A third type of RAS (intracellular/intracrine RAS) has been observed in some types of cells, including neurons. However, its role is still unknown. The present results indicate that in brain cells the intracellular RAS counteracts the intracellular superoxide/H_2_O_2_ and oxidative stress induced by the extracellular/paracrine angiotensin II acting on plasma membrane receptors. Activation of nuclear receptors by intracellular or internalized angiotensin triggers a number of mechanisms that protect the cell, such as an increase in the levels of protective angiotensin type 2 receptors, intracellular angiotensin, PGC-1*α* and IGF-1/SIRT1. Interestingly, this protective mechanism is altered in isolated nuclei from brains of aged animals. The present results indicate that at least in the brain, AT1 receptor blockers acting only on the extracellular or paracrine RAS may offer better protection of cells.

The ‘classical’ renin–angiotensin system (RAS) is a circulating humoral system that controls blood pressure. The actions of angiotensin II (AII), the most important effector peptide in RAS, are mediated by two main cell receptors: AII type 1 and 2 (AT1 and AT2). AT1 receptors mediate major effects of the system, and it is generally considered that protective AT2 receptors antagonize the pro-oxidative effects of AT1 receptors.^1^ More recently, local or tissue RAS has been identified in a variety of tissues, including the central nervous system.^2^ It is now known that the local brain RAS is involved in different brain functions, and also appears to be altered in some disorders.^3, 4^ In previous studies, we have demonstrated the presence of a local RAS in the substantia nigra pars compacta (SNc) and striatum of rodents and primates, including humans.^5, 6, 7^ This local RAS modulates dopamine release^8, 9^ possibly via mutual regulation between dopamine and angiotensin receptors.^10, 11, 12^ However, dysregulation of these interactions exacerbates neuroinflammation, oxidative stress and dopaminergic neuron death.^13, 14^ In addition, immunohistochemical studies have revealed an apparent intracellular localization of several RAS components in different types of cells, including dopaminergic neurons and glial cells of mammals, including non-human primates and human.^5, 15, 16^ However, the role of the intracellular RAS, and particularly the nuclear components of the RAS, is still unknown. In the present study we investigated the presence and possible role of major RAS components in brain cell nuclei. In particular, we investigated the possible effects of nuclear angiotensin receptors in the transcription of other components of the intracellular RAS and of several proteins that act as major regulators of the mitochondrial function. Our experiments were carried out in rats, knockout (KO) and transgenic mice, as well as in the MES 23.5 dopaminergic neuron cell line. A major difficulty in elucidating the role of intracellular RAS is to separate the responses induced by intracellular AII through intracellular or nuclear receptors from those induced by extracellular AII through activation of cell surface receptors. To overcome this technical difficulty, we investigated the effect of AII both in cells and in isolated nuclei. Our results show that nuclear angiotensin receptors control key events for nuclear–mitochondrial interaction and neuronal survival.

## Results

### Localization of AT1 and AT2 receptors in the nucleus of dopaminergic neurons

**Figure 1:**
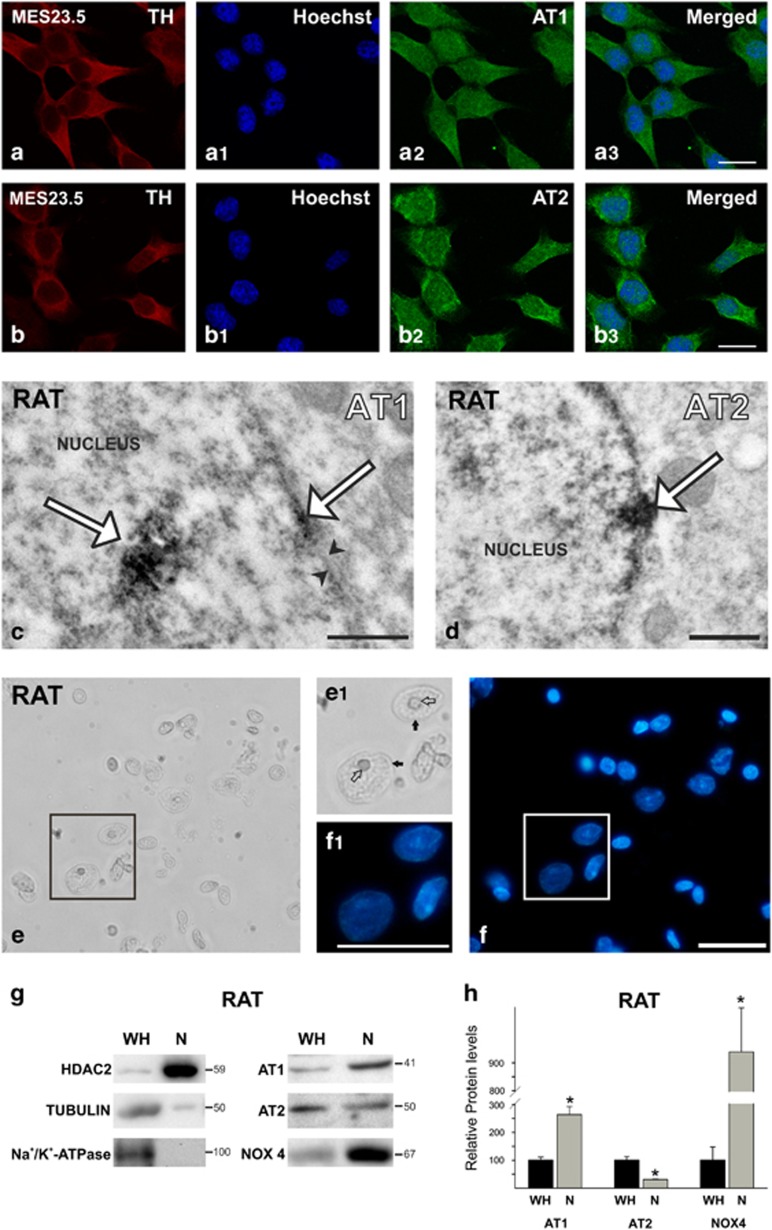
Nuclear AT1, AT2 receptors and NOX4 in nuclei from MES 23.5 dopaminergic neurons and rat nigral region. (**a** and **b**) MES 23.5 dopaminergic neurons showing triple immunolabeling for the dopaminergic marker (TH), the nuclear marker (Hoechst), and AT1 (**a**) or AT2 (**b**) receptors. (**c** and **d**) Electron microscopy of AT1 and AT2 labeling (white arrows) in nuclei and nuclear membranes (between black arrowheads) of rat dopaminergic neurons. (**e** and **f**) Nuclei isolated from the rat nigral region in the ventral mesencephalon; the integrity of nuclei was confirmed by microscopic examination with phase contrast (**e**) and Hoechst staining (**f**); areas boxed in (**e** and **f**) are magnified in (**e**1 and **f**1), respectively. (**g**) WB of whole homogenate (WH) and isolated nuclei (N) from the nigral region showing the expression of AT1 and AT2 receptors, Nox4, as well as different compartment markers used to assess the purity of the nuclei isolation (HDAC2 as a nuclear marker; tubulin as a cytosol marker; and Na^+^/K^+^-ATPase as plasma membrane marker). Note the higher expression of AT1 and lower expression of AT2 in the nucleus compared to receptors in WH (**g** and **h**). Data are mean±S.E.M. *P<0.05 compared to WH. Student’s *t-*test (*n*=3–4). Scale bars: 150 *μ*m (**a** and **b**), 0.5 *μ*m (**c** and **d**) and 50 *μ*m (**e** and **f**)

Our previous immunohistochemical and laser confocal microscopy studies have revealed immunolabeling for AT1, AT2 receptors and angiotensinogen in the nuclei of SNc dopaminergic neurons and MES 23.5 dopaminergic neuron cell line.^5^ In the MES 23.5 dopaminergic neuron cell line, labeling for AT1 and AT2 receptors also colocalized with the nuclear marker Hoechst 33342 (Figures 1a and b). Samples for electron microscopy were obtained from the densely packed dopaminergic cell clusters of the rat SNc, where immunoelectron microscopy confirmed the labeling for both AT1 and AT2 receptors in the nuclear membranes of dopaminergic neurons (Figures 1c and d).

Nuclei were isolated from the rat nigral region in the ventral mesencephalon (Figures 1e and f), and the quality of the isolation was demonstrated by the absence of markers of the cytosol fraction such as *α*-tubulin, and membrane markers such as Na^+^/K^+^-ATPase. In addition, the presence of the nuclear fraction was confirmed with the use of the nuclear marker histone deacetylase 2 (HDAC2; Figure 1g). The two main angiotensin receptor types (i.e., AT1 and AT2) were observed in the nuclear fraction. There was a clear difference in the abundance of each receptor subtype; the AT2 receptor appeared less abundant in nuclear than in total cell homogenates, while the opposite occurred for the AT1 receptor (Figures 1g and h). We also observed the presence of Nox4, which is a major cell source of intracellular ROS in the nuclear fraction (Figures 1g and h).

### Effects of AII on nuclear RAS components

**Figure 2:**
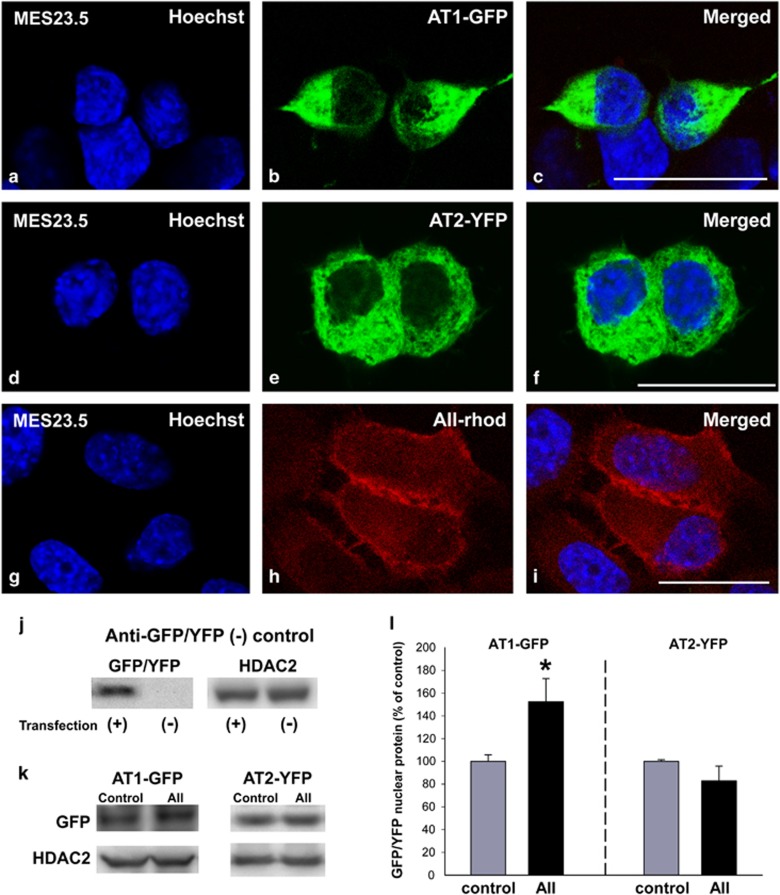
Presence of fluorescence-tagged angiotensin receptors and fluorescent AII in nuclei. Colocalization (**c**,**f** and **i**) of the fluorescent nuclear marker Hoechst (**a**,**d** and **g**) with AT1-EGFP (**b**), AT2-YFP (**e**) or AII-Rhod (**h**). WB analysis of GFP-/YFP-tagged protein in MES 23.5 transfected (+) and not transfected (−) cells showing the specificity of the common anti-GFP/YFP antibody (**j**). Treatment of MES 23.5 dopaminergic neuron cell line with AII increased nuclear AT1-EGFP receptor protein but not nuclear AT2-YFP receptor protein relative to nuclei of control cells (**k** and **l**). Data are mean±S.E.M. *P<0.05 compared to control. Student’s *t*-test (*n*=3–4). AT2-YFP, AT2 tagged to YFP; AT1-EGFP, AT1 tagged to EGFP; AII-Rhod, rhodamine-fluorescent AII. Scale bar: 20 *μ*m (**a**–**i**)

Twenty-four hours after transient transfection of the MES 23.5 dopaminergic neuron cell line, AT1 and AT2 receptors labeled with fluorescent proteins (enhanced green fluorescent protein, EGFP; yellow fluorescent protein, YFP) were located both at the plasma membrane and intracellularly, and they colocalized with the nuclear marker Hoechst 33342 (Figures 2a–f). In a second series of experiments, cultures of the MES 23.5 dopaminergic neuron cell line were treated with rhodamine-conjugated AII to investigate the localization of AII within the nucleus. The fluorescent AII was internalized and colocalized with the nuclear marker Hoechst 33342, which was evident after 30 min, and persisted for 24 h after treatment (Figures 2g–i).

Interestingly, treatment of the MES 23.5 dopaminergic neuron cell line with 100 nM AII increased nuclear AT1-EGFP fluorescence in these cells relative to nuclei from control cells. However, the same treatment did not induce any significant increase in the levels of nuclear AT2-YFP fluorescence relative to control cells. These effects were confirmed by western blot (WB) in samples from isolated nuclei (Figures 2j–l).

**Figure 3:**
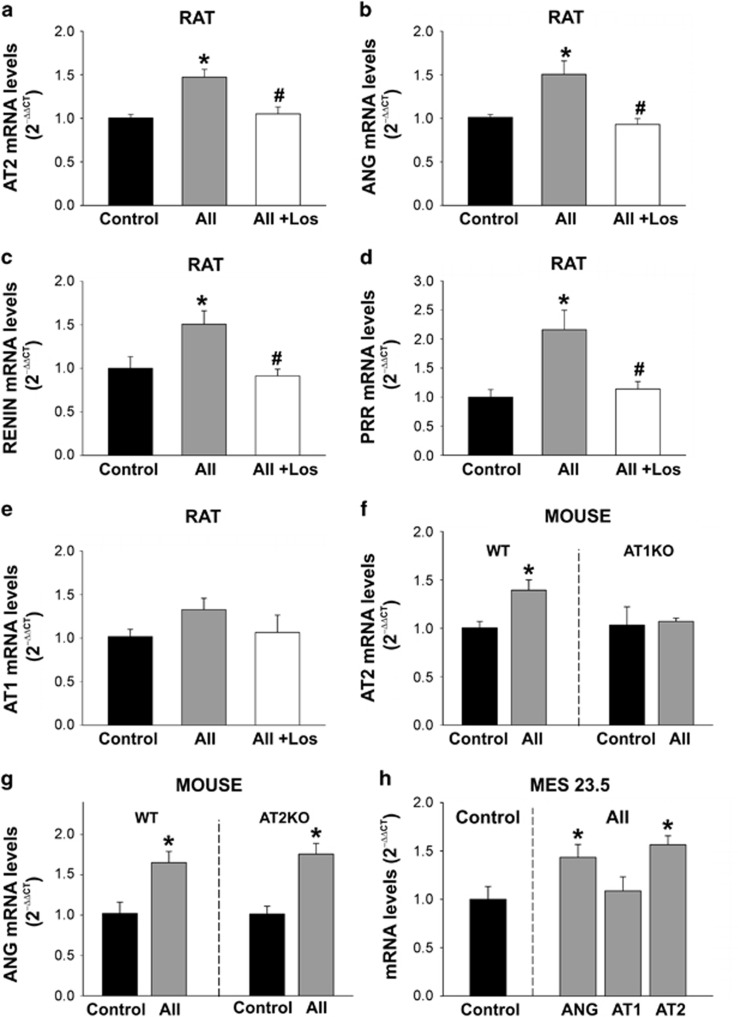
Effect of angiotensin (AII) on transcription of different RAS components. Treatment of rat-isolated nuclei with AII induced an increase in the expression of mRNA for AT2 (**a**), angiotensinogen (ANG; **b**), renin (**c**) and PRRs (**d**), which was inhibited by simultaneous treatment with the AT1 receptor antagonist losartan (los), indicating that these effects are mediated via nuclear AT1 receptors. In contrast, the levels of AT1 receptor mRNA did not change significantly after AII administration (**e**). The role of AT1 receptors in these effects was confirmed in isolated nuclei from AT1 and AT2 KO mice treated with AII (**f** and **g**). The effects of AII on brain-isolated nuclei were also observed in nuclei from the MES 23.5 dopaminergic neuron cell line (**h**). Data are mean±S.E.M. *P<0.05 compared to control, ^#^*P*<0.05 compared to the group treated with AII. One-way analysis of variance and Holm–Sidak *post hoc* test (**a**–**e**) and Student’s *t*-test (**f**–**h**) (*n*=4–8)

RT-PCR analysis of brain-isolated nuclei revealed that treatment with 100 nM AII induces a significant increase in angiotensinogen and AT2 receptor mRNA expression (Figures 3a and b), as well as an increase in the expression of mRNA for renin and prorenin/renin receptors (PRRs; Figures 3c and d). In contrast, the levels of AT1 receptor mRNA did not change significantly (Figure 3e). The increases in mRNA expression induced by All for the abovementioned RAS components were inhibited by simultaneous treatment with the AT1 receptor antagonist losartan, which indicates that these effects are mediated via nuclear AT1 receptors. This was confirmed by treatment of isolated nuclei from AT1 and AT2 KO mice. AII induced the abovementioned effects in AT2 KO, but not in AT1 KO mice (Figures 3f and g). The effects of AII on brain-isolated nuclei were confirmed in nuclei from the MES 23.5 dopaminergic neuron cell line, in which AII treatment also increased mRNA expression for AT2 receptors and angiotensinogen, but not mRNA expression for AT1 receptors (Figure 3h).

### Effects of AII on the transcription of PGC-1*α*, IGF-1 and SIRT1 in isolated nuclei

**Figure 4:**
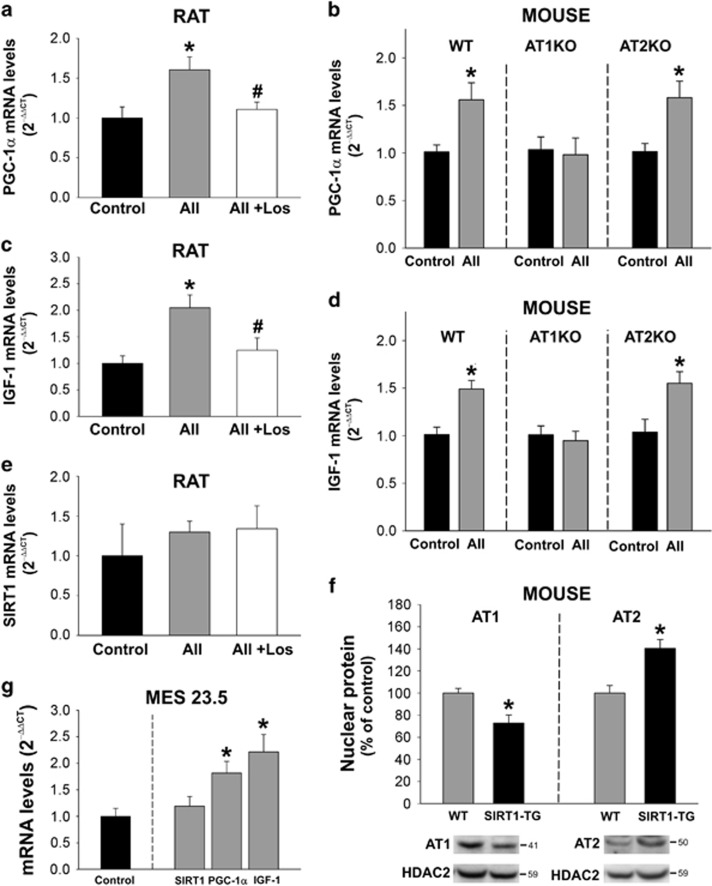
Effect of angiotensin (AII) on transcription of PGC-1*α*, IGF-1 and SIRT1. Treatment of isolated nuclei with AII induced an increase in the expression of mRNA for PGC-1*α* (**a**) and IGF-1 (**c**), which was inhibited by the simultaneous treatment with the AT1 receptor antagonist losartan (los), indicating that these increases are mediated via nuclear AT1 receptors. This was confirmed by treatment of isolated nuclei from AT1 and AT2 KO mice with AII (**b** and **d**). The expression of SIRT1 mRNA did not change significantly after treatment of isolated nuclei with AII (**e**). However, nuclei isolated from mice overexpressing SIRT1 (SIRT1-TG) showed a decrease in the expression of nuclear AT1, and an increase in the levels of AT2 receptors (**f**). The effects of AII on brain-isolated nuclei were also observed in nuclei from the MES 23.5 dopaminergic neuron cell line (**g**). Data are mean±S.E.M. *P<0.05 compared to control or WT (**f**), ^#^*P*<0.05 compared to the group treated with AII. One-way analysis of variance and Holm–Sidak *post hoc* test (**a**, **c** and **e**), and Student’s *t*-test (**b**, **d**, **f** and **g**) (*n*=4–8)

Treatment of isolated nuclei with 100 nM AII led to an ~60% increase in mRNA expression for PGC-1*α* and a twofold increase in mRNA expression for IGF-1. These increases were inhibited by the simultaneous treatment with the AT1 receptor blocker losartan, suggesting that AII acts via nuclear AT1 receptors (Figures 4a and c). When nuclei isolated from KO AT1 and KO AT2 mice were treated with All the effect was observed in nuclei from KO AT2 mice, but was absent in nuclei from KO AT1 mice (Figures 4b–d).

Isolated nuclei were also treated with AII to assess its effects on mRNA expression for SIRT1. However, the increase in SIRT1 mRNA induced by All was not significant (Figure 4e). To further investigate a possible interaction between SIRT1 and the nuclear RAS we investigated the expression of AT1 and AT2 receptors in nuclei isolated from transgenic mice overexpressing SIRT1. Interestingly, these mice showed a significant decrease in the levels of nuclear AT1 receptors, but a significant increase in the levels of nuclear AT2 receptors (Figure 4f). The results observed in nuclei isolated from the brain nigral region were also observed in nuclei isolated from the MES 23.5 dopaminergic neuron cell line, where AII treatment also produced an increased mRNA expression for PGC-1*α* and IGF-1, but a nonsignificant increase in mRNA expression for SIRT1 (Figure 4g).

### Effects of AII on superoxide/H_2_O_2_, calcium and nitric oxide levels in isolated nuclei

**Figure 5:**
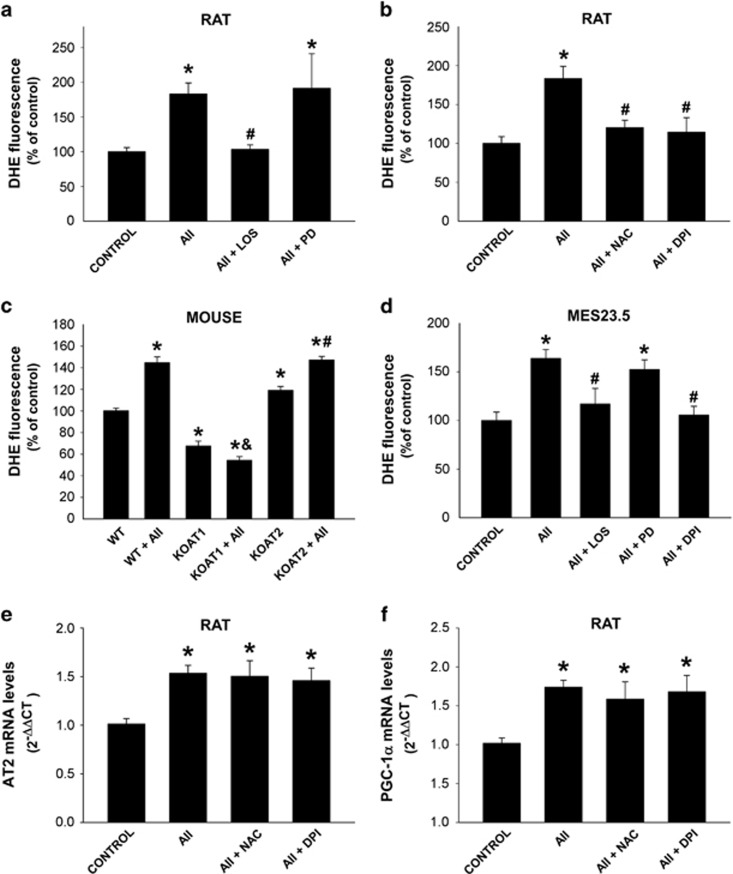
Effect of angiotensin (AII) on nuclear superoxide/H_2_O_2_ production, AT2 and PGC-1*α* mRNA expression. Treatment of isolated nuclei with AII increased the levels of nuclear superoxide/H_2_O_2_ (i.e., DHE fluorescence; **a–d**), which were inhibited by the simultaneous treatment with the AT1 receptor antagonist losartan (**a**; los), the antioxidant NAC and the Nox inhibitor DPI (**b**), but not by the AT2 receptor antagonist PD123,319 (PD) (**a**). Involvement of AT1 in these effects was confirmed by treatment of nuclei from AT1 and AT2 KO mice with AII, which also revealed an opposite effect of nuclear AT1 (increase) and AT2 (decrease) receptors on the levels of nuclear superoxide/H_2_O_2_ (**c**). The effects of AII on brain nuclei were also observed in nuclei from the MES 23.5 dopaminergic neuron cell line (**d**). However, the effects of AII on AT2 (**e**) and PGC-1*α* (**f**) mRNA expression were not inhibited by the antioxidant NAC or the Nox inhibitor DPI. Data are mean±S.E.M. *P<0.05 compared to control or WT (**c**), ^&^compared to KO AT1, ^#^*P*<0.05 compared to group treated with AII (**a**, **b** and **d**) or KO AT2 (**c**). One-way analysis of variance and Holm–Sidak *post hoc* test (*n*=4–10)

Treatment of brain-isolated nuclei with AII induced a significant increase in the levels of nuclear superoxide/H_2_O_2_, which was inhibited by simultaneous treatment with the AT1 receptor blocker losartan, but not by the simultaneous treatment with the AT2 receptor blocker PD123,319 (Figure 5a). The increase in superoxide/H_2_O_2_ induced by All was also inhibited by the simultaneous treatment with the antioxidant *N*-acetyl-cysteine (NAC) or the simultaneous treatment with the Nox4 inhibitor (diphenyleneiodonium, DPI; Figure 5b). Altogether these results suggest that at the nuclear level, AII activates the AT1/Nox4 axis to induce the release of superoxide/H_2_O_2_. The role of AT1 and AT2 receptors in AII-induced nuclear superoxide/H_2_O_2_ release was confirmed in isolated nuclei from AT1 and AT2 KO mice (Figure 5c). Levels of superoxide/H_2_O_2_ were significantly lower in nuclei from KO AT1 mice than from wild-type (WT) mice. Nuclei from KO AT2 mice showed significantly higher levels of superoxide/H_2_O_2_ than nuclei from WT mice. Treatment of nuclei from KO AT1 mice with AII induced a significant additional decrease in the levels of nuclear superoxide/H_2_O_2_ (possibly as a result of the effect of AII on nuclear AT2 receptors). Treatment of isolated nuclei from KO AT2 mice with AII induced an additional increase in the levels of nuclear superoxide/H_2_O_2_. Altogether these results reveal an opposite effect of AT1 (marked increase), and AT2 (slight decrease) nuclear receptors on the levels of nuclear superoxide/H_2_O_2_.

The observations in brain nuclei were confirmed in nuclei isolated from the MES 23.5 dopaminergic neuron cell line, in which AII treatment also induced a significant increase in the levels of nuclear superoxide/H_2_O_2_. This effect was inhibited by the simultaneous treatment with the AT1 receptor blocker losartan or the Nox4 inhibitor DPI, but not by the simultaneous treatment with the AT2 receptor antagonist PD123,319 (Figure 5d).

The AII-induced increase in AT2 receptor or PGC-1*α* mRNAs (Figures 3 and 4) was not blocked by the simultaneous treatment with the antioxidant NAC or the Nox inhibitor DPI, at least in the present experimental conditions. This suggests that the AII-induced increase in nuclear levels of superoxide/H_2_O_2_ does not mediate the abovementioned effects on transcription (Figures 5e and f).

**Figure 6:**
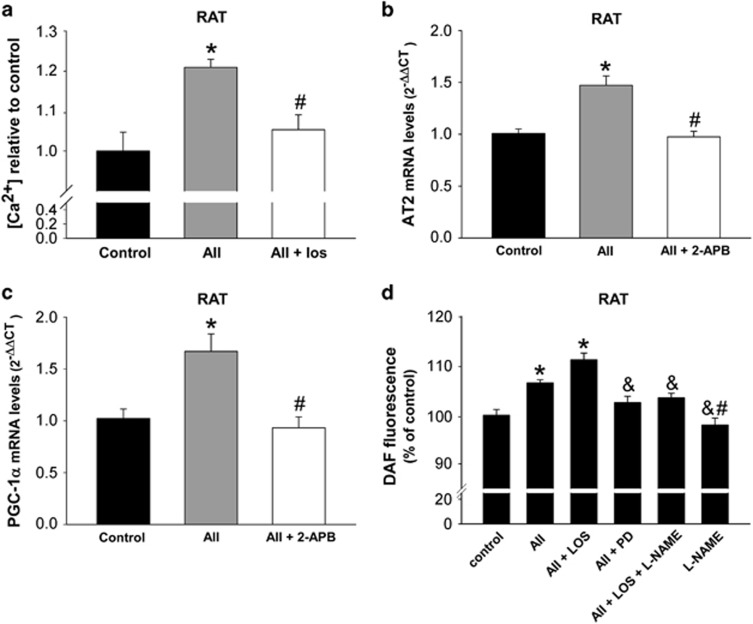
Effect of angiotensin (AII) on nuclear calcium and NO levels. Treatment of rat-isolated nuclei with AII induced a significant increase in nuclear calcium (**a**), NO levels (**d**), which were inhibited by the simultaneous treatment with the AT1 receptor antagonist losartan (los) and the AT2 receptor antagonist PD123,319 (PD), respectively. Treatment of isolated nuclei with AII also induced an increase in the expression of mRNA for AT2 (**b**) and PGC-1*α* (**c**), which was inhibited by the simultaneous treatment with the IP3 receptor blocker 2-APB, suggesting that Ca^2+^ signaling is involved in these transcriptional changes. The AII-induced increase in nuclear levels of NO was inhibited by the NOS inhibitor l-NAME (**d**). Data are mean±S.E.M. *P<0.05 compared to control, ^#^*P*<0.05 compared to the group treated with AII, ^&^compared to the group treated with AII+los. One-way analysis of variance and Holm–Sidak *post hoc* test (*n*=4–8)

Treatment of isolated nuclei with AII led to increased levels of nuclear calcium, which were inhibited by treatment with the AT1 receptor blocker losartan, indicating that the effect is mediated by AT1 receptors (Figure 6a). Interestingly, the simultaneous treatment of isolated nuclei with the IP3 receptor inhibitor 2-aminoethyl diphenylborinate (2-APB) inhibited the AII-induced increase in AT2 and PGC-1*α* mRNAs, suggesting that these effects are mediated by Ca^2+^ via nuclear IP3·receptors (Figures 6b and c).

Treatment of isolated nuclei with AII and the AT1 blocker losartan induced a significant increase in nitric oxide (NO) levels, which was inhibited by simultaneous treatment with the AT2 receptor antagonist PD123,319 (Figure 6d). This indicates that nuclear AT2 receptors mediate the AII-induced production of nuclear NO. The AII-induced increase in nuclear NO was also inhibited by simultaneous treatment with the NO synthase (NOS) inhibitor, *N*-nitro-l-arginine methyl ester (l-NAME), which indicates that the nuclear NO synthase involved in this process.

### Effects of AII on isolated nuclei from aged rats

**Figure 7:**
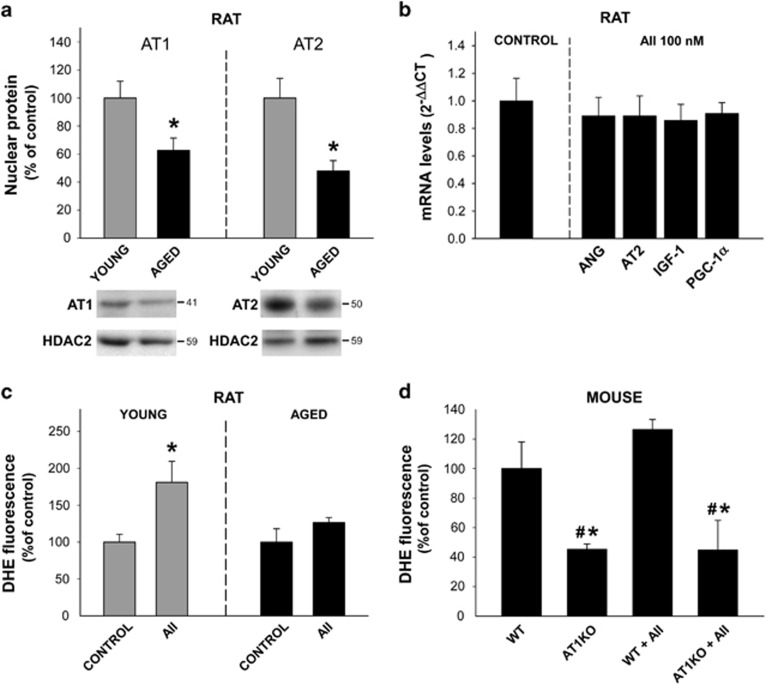
Nuclear AT1 and AT2 receptors in aged rats and mice. Nuclei isolated from brains of aged rats showed a significant decrease in the levels of both AT1 and AT2 receptors (**a**). Treatment of nuclei with AII did not induce any significant increase in AT2, angiotensinogen (ANG) and PGC-1*α* mRNA expression (**b**), or in the levels of nuclear superoxide/H_2_O_2_ (**c**). Levels of nuclear superoxide/H_2_O_2_ were significantly lower in aged KO AT1 mice than in WT aged mice, and treatment with AII did not induce any significant increase in superoxide/H_2_O_2_ in nuclei from aged KO AT1 mice (**d**). Data are mean±S.E.M. *P<0.05 compared to the corresponding control, ^#^*P*<0.05 compared to aged WT or aged WT treated with AII. One-way analysis of variance and Holm–Sidak *post hoc* test (**b** and **d**) and Student’s *t*-test (**a** and **c**) (*n*=3–8)

Nuclei isolated from brains of aged rats showed a significant decrease in the levels of both AT1 and AT2 receptors (Figure 7a). Interestingly, treatment of these nuclei with 100 nM AII did not induce any significant increase in mRNA expression for IGF-1, PGC-1*α* or AT2 receptors (Figure 7b). Similarly, the AII-induced increase in the levels of superoxide/H_2_O_2_ was not statistically significant in these nuclei (Figure 7c). As observed in nuclei from young KO AT1 mice (Figure 5c), the levels of nuclear superoxide/H_2_O_2_ were significantly lower in aged KO AT1 mice than in WT aged mice. Treatment with AII did not induce any significant increase in the levels of superoxide/H_2_O_2_ in nuclei from aged KO AT1 mice, as previously observed in young KO AT1 mice (Figure 7d).

## Discussion

**Figure 8:**
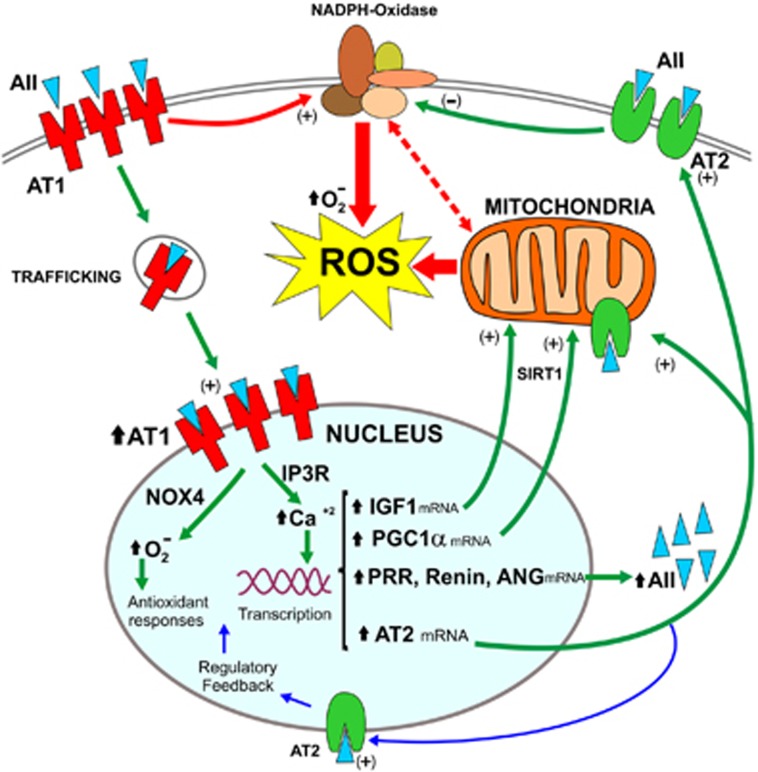
Model of the role that nuclear AT1 and AT2 receptors have in modulating the pro-oxidative effects of activation of membrane AT1 receptors by extracellular (i.e., paracrine) AII. Extracellular AII induces activation of the membrane AT1/Nox2 axis, with the generation of intracellular superoxide/H_2_O_2_ and oxidative stress (red arrows), but also induces the internalization of the AII–AT1 receptor complex to the nucleus (green arrows). Activation of nuclear AT1 receptors induces an increase in nuclear NOX4/superoxide/H_2_O_2_ and IP3/Ca^2+^ levels, which are known to regulate gene expression, triggering a number of mechanisms that may protect cells against oxidative stress (green arrows). These protective mechanisms include the following: (i) an increase in the expression of AT2 receptor mRNA, which leads to an increase in the levels of protective AT2 receptors that traffic to different cell structures such as mitochondria and membrane and induces a compensatory increase in the RAS protective arm (i.e., AII/AT2); (ii) an increase in angiotensinogen, renin and PRR mRNA, which leads to an increase in the synthesis of intracellular AII to act on intracellular AT2 receptors; (iii) an increase in mRNA expression for PGC-1*α* and IGF-1, which, interacting with SIRT1, have been related to mitochondrial protection and reduction of free radical production and oxidative damage. Nuclear AT2 receptors modulate this process (blue arrows) and counteract the effects of nuclear AT1 receptors by increasing nuclear levels of NO, which was particularly shown using isolated nuclei from AT1 and AT2 KO mice. IGF-1, insulin-like growth factor 1; IP3R, inositol 1,4,5-trisphosphate receptor; PGC-1*α*, peroxisome proliferator-activated receptor gamma coactivator 1-alpha; ROS, reactive oxygen species; SIRT1, sirtuin 1

We report here the presence of AT1 and AT2 receptors as well as Nox4 in isolated nuclei from brain cells. AT1 receptors were more abundantly expressed in isolated nuclei than in the total cell homogenate, while the opposite occurred for AT2 receptors. Interestingly, we recently observed the opposite in isolated mitochondria, where AT2 are much more abundant than AT1 receptors.^17^ Treatment of the MES 23.5 dopaminergic neuron cell line with AII induced an increase in the expression of nuclear AII and AT1 receptors. In contrast, treatment of isolated nuclei with AII induced, via AT1 activation, an increase in AT2 mRNA but not AT1 mRNA transcription. The presence of AT1 receptors in the nucleus has been observed in several types of peripheral cells, although the function of nuclear angiotensin receptors was not clarified.^18, 19^ In renal cells, a rapid internalization of the AII–AT1 receptor complex, via receptor-mediated endocytosis, has been reported.^20, 21, 22^ AT2 receptors lack a canonical nuclear localization sequence as AT1 receptors do, and generally they are not internalized following ligand activation in vascular or renal cells.^23, 24^ It has been suggested that the intracellular RAS may serve to amplify events that are initiated in the plasma membrane, leading to signal amplification in successive steps with reutilization and minimal waste.^18, 25^ Quite the opposite, the results of the present study suggest that the intracellular, and particularly the nuclear RAS, may counteract and modulate the deleterious effects of the membrane AII/AT1 activation (Figure 8). Extracellular (i.e., paracrine) AII induces activation of the membrane AT1–Nox2 axis, with the generation of intracellular superoxide/H_2_O_2_ and oxidative stress, but also induces the internalization of the AII–AT1 receptor complex to the nucleus. We observed that activation of nuclear AT1 receptors by intracellular AII triggers a number of mechanisms that may protect cells against oxidative stress. These mechanisms include an increase in the levels of AT2 receptors and intracellular angiotensin, as well as of PGC-1*α* and IGF-1. Interestingly, this protective mechanism is altered in isolated nuclei from brains of aged animals.

### Effects of nuclear AII/AT1 activation on nuclear RAS components

Activation of nuclear AT1 receptors by AII induces an increase in the expression of AT2 receptor mRNA. This may lead to an increase in the levels of protective AT2 receptors that traffic to different cell structures such as mitochondria and membrane. A compensatory increase in the opposite or protective arm of the RAS (i.e., AII/AT2) has been observed in different studies after increasing the activity of the membrane AII/AT1/Nox2/superoxide axis.^26, 27, 28^ Consistent with this, we have recently shown that AT2 receptors are particularly abundant in mitochondria and that mitochondrial AT2 receptors, via NO, downregulate mitochondrial respiration to counteract oxidative stress in neurons.^17^ In the present study, we observed that nuclear AT2 receptor activation increases the levels of nuclear NO, and our observations in AT1 and AT2 KO mice suggest that nuclear AT2 receptors modulate the effects of nuclear AT1 receptors. In addition to an increase in AT2 mRNA expression, activation of AT1 nuclear receptors by AII induced an increase in the expression of mRNA for angiotensinogen, renin and renin–prorenin receptors, which indicates that an increase in the synthesis of intracellular angiotensin may further act on intracellular AT2 receptors to counteract oxidative damage.

### Effects of nuclear AII/AT1 activation on transcription of PGC-1*α*

Activation of nuclear AT1 receptors also induced an increase in mRNA expression for PGC-1*α*. Members of the PGC-1 family have emerged as master transcriptional regulators of the mitochondrial function that improve or rescue mitochondrial dysfunction.^29, 30, 31, 32^ In dopaminergic neurons, PGC-1*α* has been shown to be essential for survival, possibly through the maintenance of mitochondrial function, as conditional PGC-1*α* KO mice show a significant loss of this type of neurons,^33, 34^ while overexpression of PGC-1*α* protects dopaminergic neurons.^33, 34, 35, 36, 37^ The present results show that activation of nuclear AT1 receptors by AII induces an increase in the expression of PGC-1*α* that may counteract the pro-oxidative effects of activation of the surface membrane AT1 receptor. Nuclear AT2 receptors modulate this process by counteracting the effects of nuclear AT1 receptors.

### Effects of nuclear AII/AT1 activation on transcription of IGF-1 and SIRT1

We also investigated the effect of activation of nuclear AT1 receptors on two additional factors (i.e., IGF-1 and SIRT1) that have been shown to modulate mitochondrial function and to interact with the RAS in studies performed at tissue and cellular levels.^38, 39^ IGF-1 has been found to increase survival of dopaminergic neurons,^40, 41, 42^ and activation of nuclear AT1 receptors increases the expression of IGF-1 mRNA. The cytoprotective effects of IGF-1 have been related to mitochondrial protection, leading to a reduction of free radical production, oxidative damage and apoptosis.^43, 44^ IGF-1 has been suggested to inhibit cell death triggered by oxidative stressors via increasing expression of SIRT1.^45^

SIRT1 is present in the nucleus and cytoplasm. It deacetylates several proteins involved in cell survival, metabolism and stress response, and stimulates resistance to oxidative stress in several types of cells.^46^ Several studies lead to the conclusion that SIRT1 exerts its control over mitochondrial function mainly by regulating PGC-1*α* activity.^30, 31, 47^ In a recent study, we observed that AII induced an increase in SIRT1 expression in dopaminergic neurons and microglia.^38^ However, the activation of AT1 nuclear receptors in isolated nuclei did not induce any significant increase in SIRT1 mRNA. This suggests that certain components located in the cytoplasm are involved in the increase in expression of SIRT1 induced by membrane AII/AT1 activation. The abovementioned increase in expression of IGF-1 may be a possible mechanism, as it has been observed that IGF-1 induced an increase in SIRT1 expression in cardiomyocytes.^45^ The involvement of nuclear angiotensin receptors in the regulation of SIRT1 levels is suggested by our observations in isolated nuclei from transgenic mice overexpressing SIRT1. Overexpression of SIRT1 led to a decrease in expression of nuclear AT1 receptors and an increase in expression of AT2 nuclear receptors, which suggests a possible feedback regulation of the abovementioned mechanism. This is also consistent with observations showing that overexpression of SIRT1 in muscle cells downregulates PGC-1*α*.^48^ In summary, activation of nuclear AT1 receptors by AII may trigger a compensatory mechanism by increasing both PGC-1*α* and SIRT1 via IGF-1.

### Effects of nuclear AII/AT1 activation on nuclear calcium and superoxide/H_2_O_2_ levels

We have observed that activation of nuclear AT1 receptors induces an increase in nuclear superoxide/H_2_O_2_ as well as in Ca^2+^ levels, which have been shown to activate signaling pathways that influence gene expression in different types of cells. Nuclear Ca^2+^ signaling is an important regulator of gene transcription,^49^ and IP3 receptors are involved in Ca^2+^ signaling.^50^ Calcium has been shown to directly bind transcription factors like DREAM,^51^ or activate the nuclear CaM kinase pathways that regulate gene expression.^52^ In the present study inhibition of nuclear IP3 receptors inhibited the AII-induced increase in AT2 and PGC-1*α* mRNAs, which suggests that Ca^2+^ signaling is involved in these transcriptional changes.

It has also been suggested that Nox4 regulates gene expression in a manner dependent on regulatory DNA sequence Maf-recognition element, constituting part of the antioxidant response.^53^ Unexpectedly, the simultaneous treatment of isolated nuclei with AII and the antioxidant NAC or the Nox inhibitor DPI did not inhibit the increase in expression of AT2 or PGC-1*α*, at least in the conditions used in the present experiments. Nox-derived superoxide/H_2_O_2_ may be involved in the transcription of other factors of the antioxidant response, possibly related to hormetic adaptations to oxidative stress similar to those described in mitochondria.^54^

### Role of nuclear RAS in aging

Aging is the highest risk factor for neurodegenerative diseases, particularly PD. In previous studies we observed that aged rats show an increase in the activity of the AII/AT1/Nox2 axis,^10, 55, 56^ which leads to increased levels of oxidative stress and neuroinflammatory markers in the nigral region. Aged animals showed an important increase in the expression of AT1 receptors, as well as a marked decrease in the expression of AT2 receptors in brain homogenates (particularly nigral and striatal homogenates). However, aged rats also showed a decrease in the levels of IGF-1 and SIRT1 in the nigral region.^39, 38^ The present findings explain these apparent paradoxical differences between young and aged rats. Nuclei from aged rats showed a significant decrease in the levels of both nuclear AT1 and AT2 receptors, and treatment of aged nuclei with AII did not induce any significant increase in the levels of nuclear superoxide/H_2_O_2_, or in the expression of AT2, angiotensinogen, IGF-1 or PGC-1*α* mRNA. This suggest that the above-described nuclear compensatory response triggered by nuclear AT1 receptors is impaired in aged cells, which leads to the decrease in AT2, IGF-1 and SIRT1 expression observed in aged animals. In a recent study, we observed that the expression AT2 receptors is decreased in mitochondria of aged rats, which may affect mitochondrial protection against oxidative stress.^17^ Deterioration of mitochondrial function and biogenesis has been postulated as a central landmark of aging, which has been related to a decline in PGC/mitochondrial function together with a decreased expression of PGC-regulated oxidative defense mechanisms.^47^

## Conclusions

The present results, and the results of our recent study,^17^ suggest that the intracellular RAS may buffer the pro-oxidative effects of activation of membrane AT1 receptors by extracellular (paracrine) AII. Internalization of the AII–AT1 complex to the nucleus, and activation of nuclear AT1 receptors by intracellular AII triggers a number of mechanisms that may protect cells against oxidative stress. Interestingly, this protective mechanism is altered in nuclei from brains of aged animals. Previous studies in non-neural cells have suggested that the intracellular RAS may serve to amplify events that are initiated in the plasma membrane. On this basis, it was assumed that intracellular AII contributes to disease progression, and that AT1 receptor blockers that are effective against extracellular and intracellular receptors, or the recent renin inhibitors that act at extra- and intracellular levels may be more effective to combat the effects of RAS overactivity on different tissues. Interestingly, it has been observed that some AT1 blockers such as candesartan act mostly on surface receptors, and other AT1 blockers such as losartan act both on surface and intracellular receptors.^57^ The present results indicate that at least in the brain, AT1 receptor blockers acting only on the extracellular or paracrine RAS may offer better protection of cells.

## Materials and methods

### Experimental design

In a first series of experiments brain sections from the substantia nigra of adult male Sprague–Dawley rats, and cultures of the MES 23.5 dopaminergic cell line were used to investigate the presence of AII receptors in the nuclei of dopaminergic cells, using immunofluorescence and electron microscopy. All experiments were carried out in accordance with the Directives 2010/63/EU and 86/609/CEE, and were approved by the corresponding ethics committee at the University of Santiago de Compostela. Animals were housed at constant room temperature (RT; 21–22 °C) and 12 h light/dark cycle. All surgeries were performed under ketamine/xylazine anesthesia. In a second series of experiments, isolated nuclei from rat nigral region were used to confirm the expression of AII receptors using WB assay. In addition, dopaminergic cells were transfected with fluorescent-tagged AT1 and AT2 receptors, or treated with fluorescent AII to investigate the colocalization of fluorescence with nuclear markers, and to confirm the results observed with immunolabeling.

For functional studies, we used nuclei isolated from the brain of young (8–10 weeks old) and aged (18–20 months old) Sprague–Dawley rats, as well as young (8–10 weeks old) and aged (18–20 months old) mice, and the MES 23.5 dopaminergic neuron cell line (generously provided by Dr. Wei-Dong Le). The following groups of mice were used: (1) male WT C57BL-6 mice (Charles River, L’Arbresle, France), and young and aged homozygous C57BL-6 mice deficient for AT1a (the major mouse AT1 isoform, and the closest murine homolog to the single human AT1; Jackson Laboratory, Bar Harbor, ME, USA); and (2) young and aged homozygous C57BL-6 mice deficient for AT2 receptors (gift of Dr. Daniel Henrion). A third group of C57BL-6 mice comprised young and aged transgenic mice in which SIRT1 is moderately overexpressed under its own promoter, thereby following the physiological pattern of expression,^58^ and the corresponding WT controls. Nuclei were incubated with AII (100 nM, Sigma, St. Louis, MO, USA) and different blockers to assess the effect of activation of AT1 and AT2 nuclear receptors on mRNA transcription, nuclear superoxide/H_2_O_2_ levels, NO or calcium levels. The blockers used included the AT1 antagonist losartan (3 *μ*M, Sigma), the AT2 antagonist PD123,319 (1 *μ*M, Sigma), the Nox inhibitor DPI (5 *μ*M Sigma), the antioxidant NAC (0.5 mM, Sigma) and the inhibitor of inositol 1,4,5-trisphosphate receptor 2-APB (50 *μ*M, Sigma).

### Inmunofluorescent labeling

Double immunofluorescence labeling was performed to identify the cells that expressed angiotensinogen/angiotensin, AT1R, AT2R and PRR in the rat SNc, as described in our previous studies.^5, 7^ Angiotensinogen/angiotensin, AT1R, AT2R and PRR antibodies were combined with antibodies against tyrosine hydroxylase (TH; as a marker of dopaminergic neurons), glial fibrillary acid protein (a marker of astrocytes) and OX42 (a marker of both resting and reactive microglia (not shown; see ref. 5 for details).

The MES 23.5 dopaminergic neuron cell line was cultured in DMEM/F12 containing Sato’s component growth medium supplemented with 2% FBS, 100 U/ml penicillin and 100 *μ*g/ml streptomycin, at 37 °C in a humidified CO_2_ incubator (5% CO_2_, 95% air). MES 23.5 cells were plated at a density of 0.4 × 10^6^ onto 35 mm plastic dishes with glass coverslips previously coated with poly-l-lysine (Sigma; 10 mg/ml), fixed in 4% paraformaldehyde in Dulbecco’s phosphate-buffered saline (DPBS; pH 7.4) for 20 min, and incubated overnight with the anti-AT1 (sc-31181; 1:50) or anti-AT2 (sc-9040; 1:200) polyclonal antibodies (both from Santa Cruz Biotechnologies, Dallas, TX, USA). After rinsing with DPBS, cells were incubated for 150 min with the Alexa-conjugated secondary antibody (Molecular Probes, Eugene, OR, USA) and then stained with Hoechst (10 *μ*g/ml; Sigma). Labeling was visualized using a confocal laser-scanning microscope (AOBS-SP5X; Leica Microsystems, Heidelberg GmbH, Mannheim, Germany). Confocal images were obtained by sequential scan using three different laser lines to avoid simultaneous excitation and possible overlap. Colocalization analysis was subsequently performed with the captured images to detect double-labeled cells. Analysis of the photographs at central cell levels revealed the location of labeling (peripheral ring suggesting membrane, cytoplasm or in the nucleus). In negative controls, in which the primary antibody was omitted, no immunostaining was observed.

### Inmunoelectron microscopy

Adult male Sprague–Dawley rats were deeply anesthetized with a mixture of ketamine/xylazine and perfused with saline (0.09% NaCl) followed by a solution of 4% paraformaldehyde and 0.1% glutaraldehyde in 0.1 M phosphate buffer (PB, pH 7.4) After perfusion, brains were quickly removed from the skull, immersed in the same fixative solution overnight at 4 °C and rinsed thoroughly in PB. Free-floating 40 *μ*m coronal sections were obtained on a vibratome and stored in PB until use. Sections containing the substantia nigra were transferred to citrate buffer, pH 6.0 (Vector Laboratories, Burlingame, CA, USA) at RT for 5 min and then to citrate buffer at 80 °C for 30 min. Sections were rinsed consecutively in citrate buffer and phosphate-buffered saline (PBS; pH 7.4) at RT before pre-treatment with 1% sodium borohydride in PBS for 15 min. Sections were rinsed several times in PBS, then transferred to a solution of 5% hydrogen peroxide in PBS for 30 min and rinsed several times in PBS. Sections were pre-incubated in 10% normal serum containing 0.01% Triton X-100 in PBS for 1 h and then incubated for 72 h at 4 °C with the appropriate primary antibody: mouse monoclonal anti-TH (Sigma; 1:10000); AT1 goat polyclonal diluted 1:100; or AT2 rabbit polyclonal diluted 1:200 (Santa Cruz Biotechnologies catalog number sc-31181 and sc-9040, respectively). Sections were rinsed thoroughly in PBS and incubated for 1 h with the appropriate secondary antibodies (biotinylated goat anti-rabbit or horse anti-goat, Vector Laboratories) diluted 1:400 in PBS containing 0.01% Triton X-100, rinsed in PBS and incubated with an avidin–biotin complex kit (1:100; Vector Laboratories) for 1 h at RT. Sections were rinsed multiple times in PBS, developed using a 3,3′-diaminobenzidine peroxidase kit (Vector Laboratories) and further rinsed in PBS.

Sections immunolabeled for AT1, AT2 or TH were rinsed in PB, transferred to a solution of 1% osmium tetroxide in PB for 1 h at RT and rinsed in PB before gradual dehydration in 50–70% ethanol. They were then transferred to a contrast solution containing 1% uranyl acetate in 70% ethanol for 1 h at RT, rinsed in 70% ethanol to remove excess uranyl acetate and gradually dehydrated in 5 min baths of 70–100% ethanol. Sections were cleared in propylene oxide and gradually infiltrated with Epon resin by sequential immersion in a 2 : 1 mixture of propylene oxide and Epon resin (30 min), 1:1 mixture of propylene oxide and Epon resin (1 h), 1:2 mixture of propylene oxide and epon (1 h) and finally transferring the sections to Epon resin overnight at 4 °C. The following day sections were transferred to freshly prepared Epon resin for 1 h at RT, flat embedded and allowed to polymerize for a minimum of 72 h at 60 °C. After flat embedding was completed, the substantia nigra was clearly identified using a bright-field microscope and re-dissected for ultramicrotomy. Semi-thin (1 *μ*m thick) and ultrathin (90 nm thick) sections were cut using a Leica EM UC6 ultramicrotome (Leica Microsystems, Wetzlar, Germany). Ultrathin sections were placed on copper grids and observed and photographed using a Hitachi transmission electron microscopy (Hitachi, Tokyo, Japan) equipped with a Hamamatsu Orca digital camera (Hamamatsu, Hamamatsu, Japan).

### WB analysis

Whole homogenates and nuclear protein extracts from the nigral region were processed for WB analysis. Equal amounts of protein were separated by 5–10% Bis-Tris polyacrylamide gel, and transferred to nitrocellulose membranes. Membranes were incubated overnight with primary antibodies against AT1 (1:200; sc-31181), AT2 (1:200; sc-9040), NOX4 (1:2500; Abcam, Cambridge, England, UK, ab133303), HDAC2 (1:200; sc-56685), *α*-tubulin (1:50 000; Sigma, T5168) or Na^+^/K^+^-ATPase (1:200; sc-21712). The corresponding HRP-conjugated secondary antibodies were used. Immunoreactivity was detected with an Immun-Star HRP Chemiluminescent Kit (Millipore, Madrid, Spain) and imaged using a chemiluminescence detection system (Molecular Imager ChemiDoc XRS System; Bio-Rad, Hercules, CA, USA).

### Specificity of antibodies

The specificity of the antibodies used for WB and immunolabeling studies has been established in previous studies: AT1 sc-31181;^59^ AT2 sc-9040.^60^ In addition, the specificity of the antibodies was confirmed in our laboratory by pre-adsorption with the corresponding synthetic peptide antigen. See ref. 61 for details. We also used WB analysis of lysates from HEK293 cells transfected with AT1 or AT2 tagged to fusion tail DDK (TA50011 from Origene, Rockville, MD, USA; DDK tag: DYKDDDDK). Using HEK293 cells the specificity of the antibodies was confirmed by the presence of a predominant immunoreactive band in lysates from positively transfected cells and also by the absence of this band in negative controls, which consisted of lysates of cells transfected with empty vectors. See ref. 17 for details.

### Transient transfection of AII receptors and rhodamine-conjugated AII treatment of MES 23.5 dopaminergic cell line

To study the cellular distribution of AII receptors, MES 23.5 cells were transiently transfected with 1 *μ*g AT1 (AT1/EGFP-N3) or 3 *μ*g AT2 (AT2/YFP-N1) complimentary DNA (cDNA) for 24 h using Lipofectamine LTX transfection reagent (Invitrogen, Carlsbad, CA, USA). Twenty-four hours after transfection, cells were treated (60 min and 24 h) with 100 nM AII, fixed in 4% paraformaldehyde, stained with Hoechst (10 *μ*g/ml) and observed using a confocal laser-scanning microscope (Leica SP5). The effect of AII in the nuclear distribution of receptors was confirmed by WB analysis. For WB, MES 23.5 cells plated onto 350 mm plastic dishes were transiently transfected with either cDNA (24 h) and then treated with 100 nM AII for 24 h. Nuclear proteins from control and treated cells (*n*=4) were obtained using the Nuclear Extract Kit (Active Motif, Carlsbad, CA, USA) and processed for WB analysis using antibodies against GFP/YFP (1:800; Life Technologies, Waltham, MA, USA; G10362) and HDAC2 (sc-56685; 1:800). Non-transfected cells were used as a negative control for the GFP/YFP antibody.

For the study of the cellular distribution of AII, MES 23.5 cells were grown on glass coverslips and treated with 0.5 nmol/ml rhodamine-conjugated AII (Phoenix Pharmaceuticals, Burlingame, CA, USA). After 3 h cells were fixed in 4% paraformaldehyde, stained with Hoechst (10 *μ*g/ml) and observed by confocal microscopy.

### Isolation of fresh intact nuclei

Fresh intact nuclei were isolated and purified from rat or mice brains and from the MES 23.5 cell line. Rats or mice were killed by decapitation, and brains were quickly removed. All isolation procedures were performed on ice or at 4 °C. Brains were rinsed with ice-cold isolation buffer A containing 320 mM sucrose, 3 mM MgCl_2_ and 20 mM Tris, pH 7.4. Brain pieces or MES 23.5 cells were homogenized in buffer A using a glass homogenizer. Homogenates were centrifuged at 1000 × *g* for 15 min at 4 °C. Supernatants were removed and pellets were resuspended in 4 ml buffer B (2.2 M sucrose, 1 mM MgCl_2_ and 10 mM Tris, pH 7.4) and differentially centrifuged at 60 000 × *g* for 60 min (Beckman XL-90 ultracentrifuge, Brea, CA, USA) using a swing out rotor at 4 °C. After centrifugation, the pellet containing the isolated nuclei was resuspended and washed by centrifugation in 2 ml buffer A. Protein content in isolated nuclei was determined using the Pierce BCA Protein Assay Kit (Thermo Scientific, Fremont, CA, USA). To confirm their integrity, fresh isolated nuclei were visualized without fixation on a phase-contrast microscope immediately after isolation. Nuclei were also stained on glass coverslips with the fluorescent nuclear marker Hoechst 33342 (10 *μ*g/ml, Sigma), and examined using an inverted fluorescence microscope (Nikon Eclipse TE300, Tokyo, Japan). For WB analysis, isolated nuclei were processed using the Nuclear Extract Kit to remove DNA and conserve only the nuclear proteins.

### Effects of AII on *in vitro* transcription of mRNAs

The effects of AII on mRNA transcription in isolated nuclei were studied using a standard *in vitro* RNA transcription system (Promega, Madison, WI, USA). Freshly isolated nuclei (100 *μ*g) were first stimulated with 100 nM AII for 30 min at 37 °C. To determine the possible mechanism involved in RNA transcription in response to AII stimulation, nuclei were pre-treated with AII in the presence of the AT1 receptor antagonist losartan (3 *μ*M), the antioxidant NAC (0.5 mM), the NOX inhibitor DPI (5 *μ*M) or the IP3 receptor blocker 2-APB (50 *μ*M). Pre-treated nuclei were then incubated with an *in vitro* RNA transcription system consisting of 500 *μ*M ATP, GTP and UTP; and 2 U/*μ*l RNasin in transcription buffer (Promega) at 37 °C for 1 h. After incubation, RNA was extracted using Trizol (Invitrogen) according to the manufacturer’s instructions. The concentration of RNA was estimated using a Nanoquant plate and an Infinite M200 multiwell plate reader (Tecan, Infinite M200, Salzburg, Austria). Total RNA (1 *μ*g) was reverse-transcribed to cDNA with deoxynucleotide triphosphate, random primers and Moloney murine leukemia virus reverse transcriptase (200 U; Invitrogen). Real-time PCR was performed to evaluate the relative levels of mRNA for several genes. Experiments were performed using a real-time iCycler PCR platform (Bio-Rad). GAPDH and *β*-actin were used as housekeeping genes and were amplified in parallel with the genes of interest. Data were evaluated by the delta–delta Ct method (2−ΔΔCt), where Ct is the cycle threshold. The expression of each gene was obtained as relative to the housekeeping transcripts. Forward (F) and reverse (R) primers were designed for each gene using C software (PREMIER Biosoft, Palo Alto, CA, USA) (Supplementary Table S1).

### Measurement of intranuclear ROS, calcium and NO levels

To study the role of AII receptors in nuclear ROS generation, isolated nuclei from MES 23.5 cells and rat or mice brains were assayed for ROS production using the fluorescent dye dihydroethidium (DHE; Sigma). In the presence of ROS DHE is oxidized to ethidium, which binds to DNA and stains nuclei with bright red fluorescence. Isolated nuclei (15 *μ*g) were treated with 100 nM AII alone or combined with the receptor antagonists losartan (3 *μ*M) or PD123319 (1 *μ*M), the antioxidant NAC (0.5 mM) or the NOX inhibitor DPI (5 *μ*M). The fluorescent dye DHE was added to the nuclei at a final concentration of 0.1 mM in a fresh working solution containing 1 mM NADPH, 3 mM MgCl_2_, 20 mM Tris and 320 mM sucrose, pH 7.40. Fluorescence was measured for 1 h at 37 °C (excitation/emission wavelength=535 nm/610 nm) using a fluorescent plate reader (Tecan, Infinite M200).

Calcium levels were estimated in isolated nuclei using the ratiometric calcium indicator Fura-2/AM (Molecular Probes). After isolation, brain nuclei were resuspended in 0.5 ml buffer containing 25 mM HEPES, 100 mM KCl, 2 mM K_2_HPO4 and 4 mM MgCl_2_. Fura-2/AM (5 *μ*M) was added to the nuclear suspension and incubated for 45 min at 37 °C. Nuclei were washed twice by centrifugation at 2500 × *g* for 5 min at RT to remove non-incorporated Fura-2/AM and resuspended in buffer containing 800 nM CaCl_2_. Calcium levels were estimated after treatment with 100 nM AII or AII+losartan (3 *μ*M) in a spectrofluorimeter (Perkin-Elmer, Norwalk, CT, USA). Fluorescence emission was detected at 509 nm and was expressed as the ratio of the two excitation wavelengths (340/380). Ca^+2^ concentrations were estimated by calibrating the fluorescent signal by sequential addition of 0.01% Triton and 1 mM CaCl_2_ and 4 mM EGTA to obtain the minimum fluorescence ratio. Data were normalized to the values of the control group (100%) to counteract possible variability among assays.

Nuclear NO production was estimated using the fluorescent dye 4-amino-5-methylamino-2′,7′-difluorofluorescein diacetate (DAF, Sigma). Isolated nuclei from rat brain were pre-incubated with 10 *μ*M DAF in buffer containing 140 mM NaCl, 14 mM glucose, 4.7 mM KCl, 2.5 mM CaCl_2_, 1.8 mM MgSO_4_, 1.8 mM KH_2_PO_4_ and 100 *μ*M l-arginine, pH 7.4 for 30 min at 37 °C. Nuclei were washed to remove any unbound dye and then incubated with 100 nM AII in the presence of losartan (3 *μ*M), PD123,319 (1 *μ*M), the NOS inhibitor L-NAME (50 *μ*M, Sigma) or buffer alone. DAF fluorescence was measured using an Infinite M200 multiwell plate reader (Tecan) at 488 and 510 nm wavelengths (excitation and emission, respectively). NO values were expressed as percentage of the corresponding controls.

### Statistical analysis

All data were obtained from at least three independent experiments and were expressed as mean values±S.E.M. Two-group comparisons were analyzed using Student’s *t*-test while multiple comparisons were analyzed using one-way ANOVA followed by *post hoc* Holm–Sidak test. Normality of populations and homogeneity of variances were tested before each ANOVA. Differences were considered significant at *P*<0.05. Statistical analyses were carried out with SigmaStat 3.0 (Jandel Scientific; San Rafael, CA, USA).

## Publisher’s Note

Springer Nature remains neutral with regard to jurisdictional claims in published maps and institutional affiliations.
