# Supplementary material for: The intracellular angiotensin system buffers deleterious effects of the extracellular paracrine system
Source: Cell Death Dis. 2017 Sep 7;8(9):e3044–. doi: 10.1038/cddis.2017.439 (PMC5636983; doi:10.1038/cddis.2017.439)
Supplement: Supplementary Table S1 [file cddis2017439x1.docx]

**Table S1.** Primers used in this study

| **Species and Genes** | **Forward (F) primer** | **Reverse (R) primer** |  |
| --- | --- | --- | --- |
| Rat ANG | 5´-GAGTGAGGCAAGAGGTGTA-3´ | 5´-TCCAACGATCCAAGGTAGAA-3´ |  |
| Rat AT1a | 5´-TTCAACCTCTACGCCAGTGTG-3´ | 5´-GCCAAGCCAGCCATCAGC-3´ |  |
| Rat AT2 | 5´-AACATCTGCTGA AGACCAATAG-3´ | 5´-AGAAGGTCAGAACATGGAAGG-3´ |  |
| Rat GAPDH | 5´-GCAAGTTCAACGGCACAGTCAAG-3´ | 5´-ACATACTCAGCACCAGCATCACC-3´ |  |
| Rat IGF-1 | 5´-CTGGAGATGTACTGTGCT-3´ | 5´-TACTTCCTTCTGAGTCTTGG-3´ |  |
| Rat PGC-1α | 5´-CACGACTCCTCCTCATAA-3´ | 5´-TACCAGAACACTCACTGT-3´ |  |
| Rat PRR | 5´-TTCACAACCTCTATGACTCC-3´ | 5´-CCACAGTTACCACATCTTG-3´ |  |
| Rat RENIN | 5´-CTCTATGACTCCTCGGAAT-´3 | 5´-CCACAGTTACCACATCTTG-´3 |  |
| Rat SIRT 1 | 5´-CAGAACCACCAAAGCGGAAA-3´ | 5´-CACAGCAAGGCGAGCATAA-3´ |  |
| Mouse ANG | 5´-CTGCTGGCTGAGGACAAG-3´ | 5´-CGAGGAGGATGCTATTGAGAA-3´ |  |
| Mouse AT2 | 5´-TGTAATCAGCCTAGCCATTG-3´ | 5´-CTACTTGACTTCCTGTTCTCG-3´ |  |
| Mouse GAPDH | 5´-AACGACCCCTTCATTGAC-3´ | 5´-TCCACGACATACTCAGCAC-3´ |  |
| Mouse IGF-1 | 5´-TGTGACATTGCTCTAACATATA-3´ | 5´-GTTGGAAGGCTGCTGATT-3´ |  |
| Mouse PGC-1α | 5´-GCCATTGTTAAGACCGAGAAT-3´ | 5´-TCTGCTGCTGTTCCTGTT-3´ |  |
| Rat and Mouse β-Actin | 5´-TCGTGCGTGACATTAAAGAG-3´ | 5´-TGCCACAGGATTCCATACC-3´ |  |
